# Editorial: The interplay of lipoprotein(a), systemic inflammation, and dietary patterns in predicting cardiometabolic disease outcomes

**DOI:** 10.3389/fnut.2026.1977546

**Published:** 2026-10-07

**Authors:** Keyang Zheng, Heng Zhang

**Affiliations:** 1 Beijing Nuclear Industry Hospital, Beijing, China; 2 Versiti Blood Research Institute, Milwaukee, WI, United States

**Keywords:** cardiometabolic disease, C-reactive protein, dietary patterns, lipoprotein(a), precision nutrition, risk prediction, systemic inflammation

Cardiometabolic diseases comprise a spectrum of interconnected metabolic and cardiovascular disorders that share common risk factors and pathophysiological pathways (1). Central obesity, insulin resistance, dyslipidemia, and hypertension promote chronic inflammation, endothelial dysfunction, and atherosclerosis, thereby increasing the risk of ischemic heart disease, stroke, and heart failure (2). Lipoprotein(a) [Lp(a)] occupies a distinctive position within this landscape: circulating levels are largely genetically determined, yet the magnitude of Lp(a)-associated cardiovascular risk may be influenced by the surrounding inflammatory and metabolic milieu (3, 4). The 10 studies assembled in this Research Topic extend this concept by examining how lipid biomarkers, inflammatory indices, dietary exposures, and composite risk measures jointly contribute to cardiovascular, metabolic, and cerebrovascular outcomes.

Two studies directly illustrate the value of integrating Lp(a) with modifiable biological or dietary factors. In a prospective cohort of 1,741 patients with premature myocardial infarction, Wang et al. found that elevated Lp(a) (>50 mg/dL; HR 2.27) and homocysteine (>15 μmol/L; HR 1.60) were independently associated with major adverse cardiovascular events (MACE), while concurrent elevation of both conferred substantially greater risk (HR 3.57). This additive interaction is clinically relevant because homocysteine, unlike largely genetically determined Lp(a), is a modifiable metabolite influenced by folate and B-vitamin status. These findings support a precision-prevention framework in which a relatively fixed inherited risk marker is interpreted alongside a correctable nutritional-metabolic one, as reflected in the authors' proposed “50–15” dual-threshold strategy for risk stratification.

A complementary UK Biobank study by Zhang paired Lp(a) with a modifiable behavioral exposure, tea consumption. Among patients with established coronary heart disease, tea intake showed a nonlinear association with MACE, with the lowest risk at approximately three cups per day (HR 0.828 vs. non-consumption). Lp(a) did not mediate this association, whereas the combined Lp(a)-C-reactive protein (CRP) profile appeared to modify its magnitude (interaction *p* = 0.050), with the strongest association observed in individuals with low Lp(a) and elevated CRP and no apparent benefit when both biomarkers were high. This distinction is important because a dietary exposure may influence cardiovascular risk without directly altering Lp(a), and its effects may instead depend on the underlying lipid and inflammatory environment. Notably, Lp(a) was dichotomized here at 50 nmol/L but at 50 mg/dL by Wang et al.; such unit heterogeneity remains a practical obstacle to comparing Lp(a) thresholds across studies.

Other contributions broaden the dietary and metabolic context beyond a single biomarker–nutrient pairing. Yi et al. analyzed cross-sectional data from 2,134 U.S. adults (NHANES) and 1,974 Chinese adults (Urumqi), and found that higher unprocessed beef intake was associated with higher triglyceride levels, with hypertriglyceridemia risk increasing more sharply above approximately 100 g/day; Mendelian randomization provided additional support for a potential causal relationship. Cui et al., meanwhile, cautioned against a simple “more is better” interpretation of nutritional biomarkers. Among 364 adults with preserved ejection fraction (179 hypertensive, 185 controls) undergoing cardiac magnetic resonance imaging, higher circulating DHA, EPA, and docosapentaenoic acid (DPA) levels were independently associated with impaired left atrial and ventricular strain in the hypertensive group only, with sex-specific patterns more marked in women. Although the retrospective design limits causal inference, these findings underscore the importance of dose, clinical context, fatty-acid subtype, background diet, and individual susceptibility when evaluating nutrient-related effects.

Composite lipid- and inflammation-related indices have shown prognostic value in cardiovascular cohorts (5), and several studies in this Research Topic apply them to new outcomes. Kahaer et al. identified the uric acid-to-HDL cholesterol ratio (UAHDL) as a potential marker of cardiometabolic-based chronic disease and incorporated it into a multivariable risk model. Jiang et al. found that the non-HDL-to-HDL cholesterol ratio (NHHR) was associated with incident carotid plaque and exhibited a nonlinear pattern in which risk rose steeply below an inflection point of approximately 4.1 and then plateaued. Deng et al. extended this integrative approach to the hemoglobin–albumin–lymphocyte–platelet (HALP) index, which combines nutritional, immune, hematologic, and thrombo-inflammatory information. Lower HALP was associated with higher mortality across the continuous metabolic syndrome spectrum, while the combination of high metabolic burden and low HALP identified individuals at particularly elevated risk, although the incremental contribution of HALP to discrimination was small (Δ*C* ≈ 0.002). Together, these studies suggest that metabolic burden and systemic vulnerability represent complementary dimensions of cardiometabolic risk.

Related studies extend this framework to diabetes and cerebrovascular disease. He et al. found that the triglyceride-glucose (TyG) index and the triglyceride–HDL–glucose–body index (TyHGB) outperformed both conventional anthropometric measures and other lipid-based indices in predicting incident type 2 diabetes. Huang et al. reported an association between the body roundness index and prevalent stroke, although its incremental discriminatory value beyond established risk factors was modest. Wu et al., in a dual-center cohort of adults with moyamoya disease, identified LDL cholesterol as an independent predictor of preoperative hemorrhagic risk and combined it with total cholesterol in a clinically oriented nomogram. Collectively, these findings illustrate both the promise and limitations of accessible biomarker-based tools: routine clinical measures can capture multidimensional risk, but statistical associations or modest improvements in discrimination do not necessarily translate into meaningful clinical benefit.

Several priorities emerge from this Research Topic. Future studies should incorporate repeated measurements of Lp(a), inflammatory biomarkers, dietary intake, and metabolic indices to better capture temporal changes and disease trajectories. Studies should also clearly distinguish mediation from effect modification (6), prioritize prospective designs with adjudicated outcomes over cross-sectional or single-cohort analyses, and externally validate prediction models in diverse populations. Most importantly, observational findings should inform, but not replace, randomized dietary or biomarker-targeted intervention studies.

Together, these contributions support a shift from single-factor risk assessment toward integrated cardiometabolic phenotyping. Lp(a) represents an important component of inherited atherothrombotic susceptibility, while inflammation, nutritional status, dietary exposures, and metabolic dysfunction may influence how that susceptibility translates into clinical disease. The next generation of precision nutrition research should therefore ask not only whether a food, nutrient, or biomarker is associated with disease, but also in whom, under which metabolic and inflammatory conditions, and whether modifying that context can meaningfully improve outcomes.
